# Supplementary material for: Aflatoxicosis outbreak and its associated factors in Kiteto, Chemba and Kondoa Districts, Tanzania
Source: PLOS Glob Public Health. 2023 Aug 8;3(8):e0002191. doi: 10.1371/journal.pgph.0002191 (PMC10409262; doi:10.1371/journal.pgph.0002191)
Supplement: Attachment — Submitted filename: Responses to reviewers.docx [file pgph.0002191.s001.docx]

Responses to reviewers

Dear Editor

Thank you for considering us!

Please receive our responses in red colored text. In this revision, we are also including an ethical statement and attached the certificate provided research institute.

PGPH-D-23-00013
Aflatoxicosis Outbreak and Its Associated Factors in Kiteto, Chemba and Kondoa Districts, Tanzania
PLOS Global Public Health

Reviewer #1: I think the authors made a great choice in terms of their starting point. I'm not convinced by the literature, though.
As a first step, the author should inform readers about the background of the disease, what factors contribute to its spread, and the worldwide perspective on the issue. Therefore, they should focus solely on saving the lives of Africans in imminent danger from this should be pointed. then references to the relevant literature from the region should be made to narrow the scope of this investigation.

Thank you for this important comment. The background part of the manuscript has been revised to include all of the comments.

Writers owe it to their audiences to inform them about the background of their neighborhood of choice, e.g., 4 specific districts.

Thank you for this comment. Our study is restricted to the affected districts which are Kiteto, Kondoa DC and Kondoa TC. We have provided information regarding to these districts. We have also introduced to our readers that these are the same districts that reported sporadic aflatoxicosis cases in 2016, 2017 and 2019.

Author should make connection from developed world literature to how this outbreak may have been avoided through proper storage and cleaning of food crops prior to processing, and how public health policies could have assisted in this endeavor.

Thank you for this important comment. We have responded to this comment in by modifying text in both background and discussion section. We have included situations from both developed and developing countries. In developed world, we have presented how the countries minimize the risk of aflatoxin contamination.

The author should mention these factors in the implications section because we cannot alter demography or lifestyles but can suggest policies that may lead to systemic prevention in the future.

Thank you for this important comment. We have added the factors as instructed

The discussions and conclusions are oversimplified and need to be rewritten in light of the literature and building sociological and scientific argument. Readers interested in public health from all around the world (developing, non-developing, disease-free, infected) should get something from this article.

We agree with you Sir/madam. We have improved our discussion by adding more relevant issues and references. We have included situations from both developed and developing countries.

Figure 1 is of low quality.

Thank you for the comment. We have modified figure 1 to improved clarity

Reviewer #2: Thank you for an informative manuscript dealing with a rare but important condition. However, the authors need to address the following.
Background
a) Include food crops that have been implicated in aflatoxicosis.

Thank you for this important comment. We have added the food crops that are implicated in aflatoxicosis

Results
a) What proportion of cases was identified through active search?

Thank you for the nice comment. We have added some information in the first paragraph of the RESULT section to answer your comment. 74% of the cases were identified through active search.

b) Were cases and controls similar at baseline?

Thank you for the important question. We have added some information in the first paragraph of the RESULT section to answer your comment.
